# Manteia, a predictive data mining system for vertebrate genes and its applications to human genetic diseases

**DOI:** 10.1093/nar/gkt807

**Published:** 2013-09-12

**Authors:** Olivier Tassy, Olivier Pourquié

**Affiliations:** ^1^Institut de Génétique et de Biologie Moléculaire et Cellulaire (IGBMC), CNRS (UMR 7104), Inserm U964, Université de Strasbourg, Illkirch. F-67400, France, ^2^Stowers Institute for Medical Research, Kansas City, MO 64110, USA and ^3^Howard Hughes Medical Institute, Kansas City, MO 64110, USA

## Abstract

The function of genes is often evolutionarily conserved, and comparing the annotation of ortholog genes in different model organisms has proved to be a powerful predictive tool to identify the function of human genes. Here, we describe Manteia, a resource available online at http://manteia.igbmc.fr. Manteia allows the comparison of embryological, expression, molecular and etiological data from human, mouse, chicken and zebrafish simultaneously to identify new functional and structural correlations and gene-disease associations. Manteia is particularly useful for the analysis of gene lists produced by high-throughput techniques such as microarrays or proteomics. Data can be easily analyzed statistically to characterize the function of groups of genes and to correlate the different aspects of their annotation. Sophisticated querying tools provide unlimited ways to merge the information contained in Manteia along with the possibility of introducing custom user-designed biological questions into the system. This allows for example to connect all the animal experimental results and annotations to the human genome, and take advantage of data not available for human to look for candidate genes responsible for genetic disorders. Here, we demonstrate the predictive and analytical power of the system to predict candidate genes responsible for human genetic diseases.

## INTRODUCTION

The recent explosion of bioinformatics resources provides an increasing amount of data and an expanding array of tools for researchers. Some of the databases where this information is available are specialized for a specific type of data such as expression data [e.g. Unigene (1), Gene Expression Omnibus (2), Arrayexpress (3)], genomic data [e.g. Ensembl (4), the National Center for Biotechnology Information (NCBI) (1)] or genetic disease data [e.g. the Online Mendelian Inheritance in Man database (OMIM) (5)]. Several resources have been established gathering the information relevant to specific model organism such as the Mouse Genome Informatics database [MGI (6)] and the Zebrafish Information Network database [ZFIN (7)]. However, the way the information is formatted and stored in such specialized databases greatly limits the possibilities for combined analysis of multiple data sets. The information is usually formatted for the system that hosts it, which makes it difficult to correlate and compare different data from different databases. Even in systems that specialize on a particular model organism, the information is usually compartmentalized and can only be explored individually. Conversely, the understanding of events involved in development, physiology and disease requires one to gather a wide variety of experimental observations and to study how events correlate over time. This requires not only a resource that hosts the relevant information, but also a system that provides the right tools to extract and exploit the full range of these data in a coherent environment. When we started this project, the annotation of the chicken genome was poor, yet the first microarrays were becoming available. Manteia was initially designed to be able to help annotate these microarrays by importing annotation from other species. Then the possibility to manipulate large lists of genes, probesets and other types of annotations was added together with statistical analysis modules allowing to directly analyze the enrichment in particular annotation categories (8–10). New functionalities and vertebrate species annotations were progressively added to the database as requested by investigators with the goal of providing a user-friendly tool, allowing biologists to mine their data without knowledge of bioinformatics. Manteia is designed to annotate and analyze large volumes of data by combining major resources from human and vertebrate model organisms. The system provides new tools and concepts to compare the annotation of ortholog genes and to analyze the information and make insightful testable predictions to help in the understanding of biological and developmental events as well as genetic disease etiology.

## MATERIALS AND METHODS

### Data and software

Part of the Manteia architecture is based on the Aniseed Gmod (11). The data used in Manteia are stored in a PostgreSQL8 database. The user Web site and the administration tools are written in PHP5 and use javascript and AJAX to enhance the interaction with users. Dynamic graphs are built using the D3 JavaScript library. Statistics tools use R to compute the hypergeometric test. Data originating from Ensembl are downloaded using BioMart. Affymetrix probe sets originate from the Human Genome U133 Plus 2.0 array, the Mouse Genome 430 2.0 and MoGene 1.0 st arrays, the Chicken array and the Zebrafish array. The other data are downloaded directly from their respective Web sites. The download, the processing and the storage of the information in the database are automated using the Manteia management tools. Manteia runs on every modern browser provided they are up-to-date, including Explorer, Firefox, Safari and Chrome. The results presented in this article may change as the database is updated regularly.

### Entity–Quality method implementation

The Entity–Quality (E-Q) method is used to find which terms are related in the human and mouse phenotype ontologies. The logical definitions of these ontologies (the decomposition of what is affected and how) use the Phenotype and Trait Ontology [PATO (12)] to describe the nature of the phenotype abnormality and an anatomical ontology [FMA (13) for human and MA (14) for mouse] to describe the affected organ. The correspondence between terms from FMA and MA are found using the Uber-anatomy ontology [UBERON (12)]. The logical definitions and these ontologies were downloaded from the Open Biological and Biomedical Ontologies Foundry [OBO (15)]. Terms from the human phenotype ontology (HPO) that could not be mapped to their corresponding mouse terms were annotated with the mapping of their closest annotated parent. When no correspondence can be found this way, Manteia uses a keyword system created manually to relate phenotypes affecting the same organ or function. More than 50 keywords are used. Manteia uses a lower weighting for queries using the keyword system to reflect the lack of precision of this method compared with the E-Q approach.

### OMIM prediction statistics

To compute the number of correct genes expected by chance for each ranking position when picked randomly in the genomic regions, we used the following for each rank i:


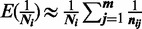
 having 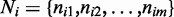
_,_ the number of regions with at least i genes, and n_i_, the number of genes in regions with at least i genes.

## RESULTS

### System presentation

Manteia is an integrative database that gathers most of the data generated for human and vertebrate model organisms in a same framework. The system is designed to be used by biologists or geneticists and does not require any specific knowledge in bioinformatics. It includes sophisticated data mining tools that allow the combination of data from different origins, nature and species. The data stored in Manteia originate from four vertebrate species: human, mouse, chicken and zebrafish. The information stored in the system covers different aspects of embryonic development and mutations/genetic disorders leading to abnormal phenotypes and diseases in animal models such as zebrafish or mouse and in humans. Data are organized into three main categories: Molecular, Expression and Developmental data, each corresponding to a different menu in Manteia. Molecular data consist of functional annotations from Gene ontology [GO (16)], chromosome location and single-nucleotide polymorphisms (SNPs) from Ensembl, protein motifs from Interpro (17), transcription factors from the DNA-binding domain database [DBD (18)] and orthology data computed from Inparanoid (19) and Compara (4) to link together the genes of different species. In addition, the data from reactome (20) are used to describe interactions, complexes and pathways, while data from the Encyclopedia of DNA Elements [ENCODE (21)] are used to predict the transcription factors regulating gene expression. Expression data comprise *in situ* hybridization from the MGI and Embrys (22) as well as expressed sequence tags (ESTs) from Unigene. EST data can be used to evaluate the expression level of genes using a digital differential display approach similar to ZooDDD (23). Embryological data include phenotype descriptions of mutations for human, mouse and zebrafish originating from OMIM, MGI and ZFIN, respectively. All these descriptions make use of ontologies including the mammalian phenotype ontology (24), the HPO (25) and the fish anatomical description. HPO is used to provide a semantic description to OMIM data.

**Figure 1.**
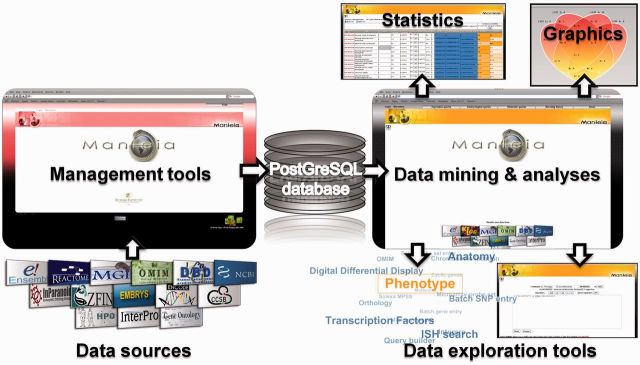
Overview of the Manteia architecture. Data are collected from various databases for several species. They are then processed by the management tools and accessed with the online user interface. Over 30 exploration, graphical representation and statistical tools are available. All the tools are interoperable, which makes it possible to analyze the information, perform data mining and make predictions.

**Figure 2.**
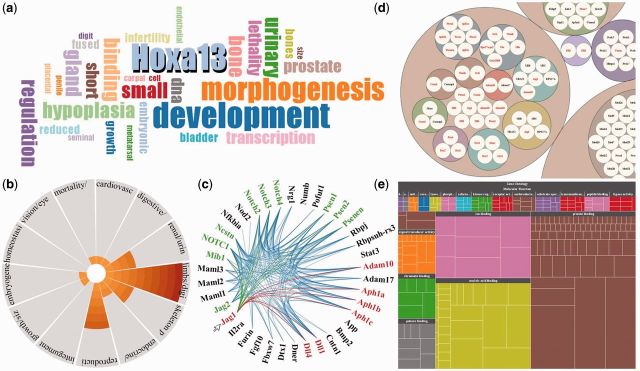
Graphical representations. Manteia uses interactive graphs to represent a result or simplify an annotation. Figure (**a**) is a word cloud computed from a gene file to give an overview of its functions. The more a word is used in the page, the bigger it appears in the cloud. (**b**) Specific annotation based on ontologies like GO or phenotypes are simplified using a radar chart showing the distribution of individual terms in broad annotation categories. A list of genes can be analyzed using dedicated graphs to represent their interactions (**c**) or the molecular complexes they form (**d**). GO, phenotypes and protein motifs annotations are represented using tree maps where each tile represents a keyword and its size the number of genes corresponding to this category.

Comparing the information related to a given gene across data sets remains a challenging task. This is due in part to the fact that the data are associated to gene models that are specific to the database they originate from. To address this problem, Manteia links all the information from the databases described above to the same set of gene models allowing an easy comparison of genes and their annotations across vertebrate species. We chose to use the Ensembl models as our reference set, but gene models from NCBI are also included and can be browsed independently in the system. Affymetrix microarray probe sets for Human, mouse, fish and chicken are linked to both sets of gene models, allowing one to maximize the information available to describe each sequence and interpret the experiments. All the data are processed automatically by the Manteia management tools (Figure 1). For each gene model, Manteia provides a page listing all the available information. The page is analyzed dynamically to generate charts designed to provide an overview of the annotation (Figure 2a and b). It is also possible to combine the data from all the orthologs and corresponding gene models so the information can be compared and completed within a same page. This way, users can easily access the most comprehensive data set available.

The Manteia system is particularly useful to analyze large lists of data such as those generated by microarray, high-throughput sequencing or proteomics analyses. It accepts inputs including lists of genes, ESTs, probe sets, SNPs and chromosome regions with the main identifiers used in the community, which can be pasted from their original files directly into the Manteia interface. Genes returned by the system can be exported with their annotation features as tabulated text files using the ‘Refine’ tool described below. Similarly, a cross-reference system allows the user to convert the gene identifiers to ensure compatibility with other databases and software.

### Data exploration tools

#### Refine

Each type of data is accessible in the system through its own dedicated interface, providing the most relevant options for each query. However, one of the strengths of Manteia comes from its ability to combine different sources of information to address complex biological questions. A particularly useful tool of the Manteia interface is called ‘Refine’ and it is designed to filter the results from a query with another tool of the system (see all the options in Table 1). For example, suppose one wishes to find which transcription factors are responsible for a muscle phenotype in mouse and are known to lead to a genetic disease in man. To that end, one can use the tool ‘transcription factor’ in the ‘Molecular data’ menu of Manteia to list all the known mouse transcription factors, then use ‘Refine’ (selecting ‘Phenotype’ and then entering the term ‘muscle phenotype’) to keep only those factors whose knockout in the mouse shows a muscle defect. Then one can obtain the list of the human orthologs of these genes using ‘Refine’ again (with the ‘Orthology’ tool) and finally use ‘OMIM’ without any parameters to list all the diseases related to these genes (see Supplementary Video S1). This ultimately leads to a list of 67 transcription factors whose knockout in the mouse gives a muscle phenotype and whose human orthologs are annotated in OMIM as associated to a human genetic disorder. Hence, all the tools of the system can be linked in a sequential manner to create a pipeline of analysis. Not only does this approach allow one to use heterogeneous data together, but it allows the transfer of annotation from one species to another. This way it is possible to predict phenotypes or diseases even in species for which these features are poorly documented like in chicken.

**Table 1.** Tools available from the ‘Refine’ interface

Expression data	
* In situ*	*In situ* hybridization expression data
Digital differential display	Identifies genes differentially expressed in different samples using ESTs
EST count	Predicts gene expression levels using ESTs
Annotations	
GO	Gene functional annotation
Protein motif	Protein motif prediction
Phenotype	Phenotype description for mutated genes
OMIM	Human genetic disorder description
Chromosome location	Returns the genes contained in a given chromosomal region
SNP	Returns the genes associated to a given single-nucleotide polymorphism
Biological pathway	Returns the genes involved in a given biological pathway
Molecular complex	Returns the genes involved in a given molecular complex
Interactome	Returns the genes involved in a given molecular interaction
Transcription factors from DBD	Identifies the genes with a transcription factor activity
Transcription targets	Returns the genes that regulate or are regulated by the given genes
Annotated with …	Returns the genes annotated with one of the data sets listed above
Species	
Orthology	Returns the corresponding orthologs of a gene
Species filter	Returns the genes that belong to a given species
Boolean tools	
Query builder	Addresses Boolean questions to the system using a mixture of data
Annotation distribution	Shows the distribution of genes in different annotation categories
Boolean list	Identifies shared or specific genes from two lists
Venn diagram	Creates a Venn diagram for up to four lists of genes
Export	
Create custom Ref for Statistics	Creates a custom reference to be used with statistics tools
Convert gene ID list	Converts a gene identifier into another identifier
Export gene ID list	Exports the current list of genes
Export annotation	Exports the annotation features of the current list of genes
Export corresponding probe sets	Exports the corresponding probe sets of the current list of genes

‘Refine’ can be used for several types of analysis. When a list of genes is provided to a tool using ‘Refine’, the user can select a keyword to extract the genes corresponding to this feature. However, when no keyword is provided, this tool analyzes all the genes from the list and displays the corresponding annotation as a text or an interactive graph (Figure 2c–e). This provides an overview of the annotation available for these genes and highlights the features they have in common.

#### Annotation enrichment and statistical analysis

**Figure 3.**
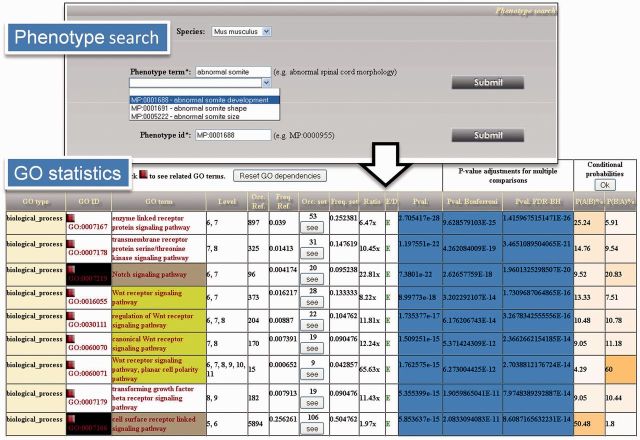
Statistics tools. The statistic module of Manteia allows one to highlight the terms of an annotation that are enriched or depleted in a set of genes. Here the GO annotation enrichment is exemplified. The *P*-value column gives the significance of the enrichment. The two following columns correct this value for multiple testing. The blue color indicates a statistical significance. Terms that are related in the ontology can be highlighted to ease the analysis. Here GO terms related to the *NOTCH* and *WNT* signaling pathways are colored in beige and green, respectively. Statistics tools can be used in combination with other exploration tools to compute a correlation between different types of data. In this example, ‘Phenotype search’ (on top) is used to assemble a list of genes leading to an abnormal somite development. The resulting GO statistics are filtered with the keyword ‘signaling pathway’. The conditional probability module (right hand side on the GO statistics screenshot) is used to compute the probability of having a gene annotated with a given GO term when it is already annotated with the ‘abnormal somite development’ phenotype (first column), and conversely (second column).

When large lists of genes or proteins from high-throughput experiments such as microarrays or proteomics are being studied together, it is often informative to analyze their annotation to identify possible enrichment in particular functional or structural categories. To that end, we designed statistics tools that can list every annotation of a group of genes and tell the user if a given category is enriched or depleted compared with a reference, for example, a genome, a microarray or a custom designed list (Figure 3). Our statistics tools work similarly to GOstat (26), extending the concept to a wider variety of data including GO, Interpro, phenotype, pathway, molecular complex and chromosome distribution. The significance of each result is given with a *P*-value computed with a hypergeometric test and corrected for multiple testing using the Bonferroni and the Benjamini-Hochberg false discovery rates. The resulting table allows the user to retrieve the list of genes associated with each annotation by a simple click, making it possible to select them specifically for further analysis. Additional features allow one to dynamically filter the results and highlight annotation categories that are related together; this greatly eases the analysis of the results. The Manteia statistics tools have been successfully used to characterize the function of cyclic genes (8), highlight the role of genes expressed in the presomitic mesoderm (9) and identify genes involved in sex determination in the chicken (10).

The scope of application of statistics tools has been extended with a conditional probability module that makes it possible to study the correlation between different annotations. For example, to identify the biological pathways involved in somitogenesis, it is possible to select the genes responsible for an ‘abnormal somite development’ using the ‘Phenotype’ search tool, then perform a statistical analysis on GO annotations and filter the GO terms related to ‘signaling pathway’ (Figure 3). The conditional probability module then shows that 15.5% of genes involved in the ‘abnormal somite development’ belong to the Wnt pathway, 10.5% to the Notch pathway and 9% to the transforming growth factor beta and bone morphogenic protein pathways. The correlation is weaker with the smoothened (4%) and the fibroblast growth factor (5%) pathways. When this statistical analysis is performed on the phenotype annotation, the same data set shows nontrivial correlations with a cardiovascular system phenotype (74%), an abnormal brain morphology (51%) or a craniofacial phenotype (52%). All these features were found significantly enriched with a false discovery rate <1%. This way the system highlights developmental events driven by the same genes and can help predict syndromic associations in human based on such associations in animal models.

### Using mouse phenotypes to predict candidate genes for human genetic diseases

**Figure 4.**
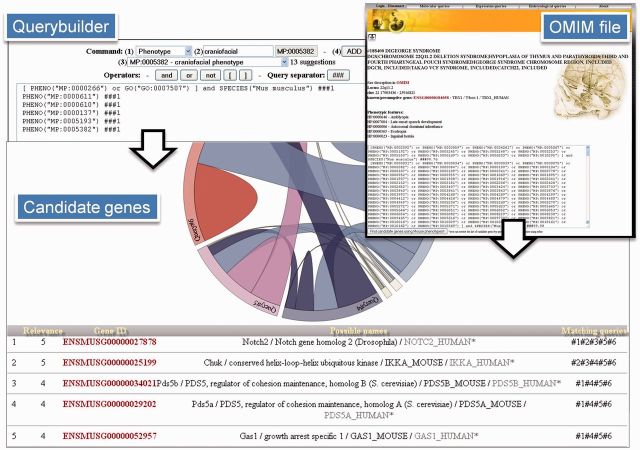
Data integration tool. The ‘Querybuilder’ makes it possible to address a question to the system using several types of data. The user enters a keyword, selects the best matching suggestion among a list and builds a question using Boolean operators (and, or, not). Several questions can be addressed at the same time using a separator followed by a weight reflecting the relative importance of each query. Alternatively, Manteia can automatically create a query from an OMIM file to look for the best candidate genes for that disease. The matching genes are ordered according to their relevance. The column at the right hand side indicates which queries match the gene annotation. Last, an interactive chord diagram is generated to show how many genes are returned by each query and how many share the same features.

We have also developed tools allowing the use of mouse annotation to identify candidate genes for specific human genetic diseases. This tool that allows to ask complex queries is called ‘Querybuilder’. It enables the user to query the database by typing a complex question (Figure 4). Questions combine the search of specific features with logical operators (and, or, not). For example, ‘[SPECIES(“*Mus musculus*”) and PATHWAY(“Signaling by NOTCH”) and GO(“nervous system development”)] not IP(“Notch domain”)’ will return all the mouse genes from the Notch signaling pathway that are involved in the nervous system development but not coding for a Notch domain. Questions to the system can take advantage of the multiplicity of information available to complement certain data with others. For example, looking for ‘[GO(“heart development”) or PHENO(“abnormal cardiac development”)] returns more results than each query ran separately.

‘Querybuilder’ offers the possibility to design several queries and have them evaluated at the same time by the system. The list of genes returned are ordered by the number of conditions they meet. Each query can be assigned a weight to reflect its importance in the analysis. This powerful approach permits one to evaluate several conditions without discarding the genes that do not meet all the criteria. Instead, a score is given reflecting the pertinence of a gene as a match to the different criteria. This last feature is a particularly powerful way to use the phenotypic data from mouse mutants to find candidate genes responsible for complex human syndromes or diseases. For example, the Holt-Oram syndrome is characterized by an abnormality of the thumb and an atrial septal defect. To look for genes responsible for this syndrome, one can design two distinct queries using the mouse phenotype terms: ‘abnormal digit morphology’ and ‘atrial septal defect’. Manteia returns 15 genes having both features including *TBX5*, which is known to lead to this disease (see Supplementary Table S1). Candidate genes can be further investigated thanks to all the data provided by the system. For instance, this query returns *SALL4* as a candidate. This gene is responsible for the Okihiro syndrome with limb defects similar to the ones found in the Holt-Oram syndrome but it also induces ocular and renal anomalies that are not found in these patients or in the animal models.

For Alagille syndrome, one can design six queries corresponding to the main features of the disease: ‘jaundice’, ‘cholestasis’, ‘abnormal cardiac morphology’, ‘abnormal vertebrae morphology’, ‘abnormal anterior eye segment morphology’ and ‘craniofacial phenotype’. Manteia returns no gene with all six features, but finds two candidates with five of them, including *NOTCH2*, which is known to be responsible for Alagille syndrome (see Supplementary Table S2). The gene that ranks second is *CHUK*. This gene shares many phenotypic features with *NOTCH2* in the mouse but has not been associated to a human disease. The second gene known to be responsible for this syndrome is *JAG1*. This gene shares four features with the mouse phenotype description and ranks within the first 30 candidates out of the entire set of mutated genes in mouse genome. Using a similar approach, ‘Querybuilder’ has been used to study the origin of the clinical features of the 2q37 deletion syndrome from the numerous genes found in this locus (27).

**Figure 5.**
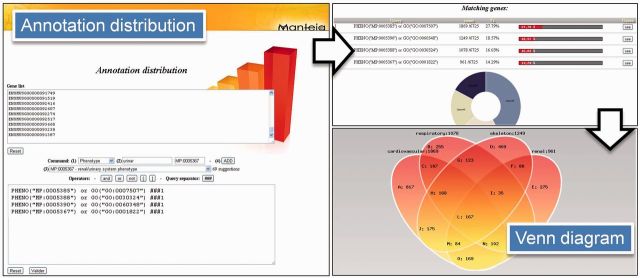
Annotation distribution. The ‘Annotation distribution’ tool generates a bar plot and a donut chart showing how many genes correspond to each Boolean query defined by the user. Here the search for genes responsible for the development or the abnormality of the cardiovascular, respiratory, skeletal and renal systems is exemplified. This way it is possible to see the relative importance of different annotation categories in a given data set and see how the distribution evolves over different experimental conditions. The Venn diagram generator can then be used to see the genes that are shared among the results returned by the queries.

Two additional customized versions of the ‘Querybuilder’ are available in the ‘Boolean tool’ section of Manteia. One is called ‘medical diagnostic’ and allows to find a genetic disease based on clinical features. The second, called ‘Annotation distribution’, counts how many genes correspond to each query defined by the user (Figure 5). This is particularly useful to analyze high-throughput experiments and quantify how many genes belong to each category (e.g. cell cycle, signaling, metabolism) and see how these values evolve over different experimental conditions.

Boolean tools can be used in combination with ‘Refine’, thereby providing unlimited ways to combine the information contained in Manteia. Altogether, these tools provide a highly sophisticated way to perform a global search using several types of data from different species.

#### Automatic candidate gene predictions for OMIM diseases

When the description of a disease is available from OMIM, Manteia is able to automatically generate the ‘Querybuilder’ question allowing the identification of the best candidate genes for that disease. To do this, the system uses the formatted description of the disease provided by HPO and searches for the equivalent terms in the mouse phenotype ontology to generate the query. This correspondence is based on an enhanced version of the E-Q (28) method (see ‘Materials and Methods’ section), which defines each phenotype in terms of what is affected and how, with the consequence of making different phenotype ontologies compatible. A weight is used, taking into account the frequency of each feature in OMIM to focus on the disease specificities. When the query is run, the system ranks the mouse candidate genes according to the number of keywords they share with the clinical description of the human disease and the weight given for each feature.

**Figure 6.**
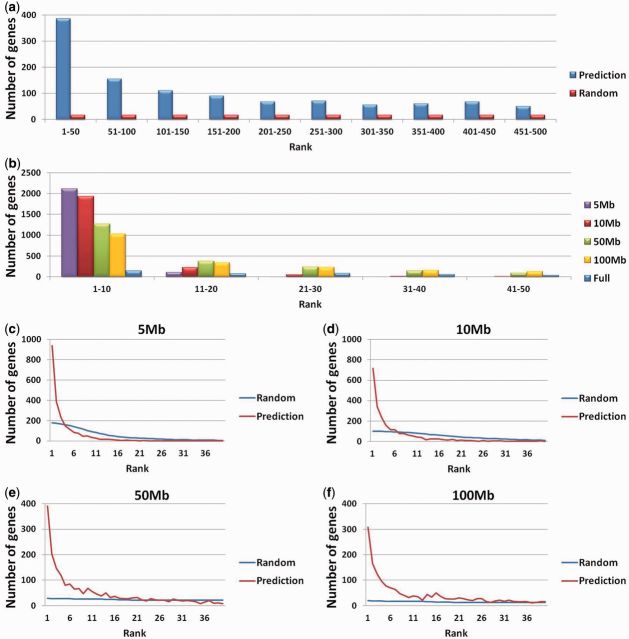
Candidate genes prediction for OMIM diseases. Manteia ranks mouse candidate genes according to the number of phenotypic features they share with the human disease genes. Figure (**a**) shows the ranking of mouse genes when searched using the E-Q method alone with the number of genes expected by chance. Figure (**b**) shows the distribution obtained when genes are searched in an area of 5 (purple), 10 (red), 50 (green) and 100 Mb (yellow). Most of known or suspected disease genes rank within the first 10 candidates. (**c–f**) shows the ranking distribution for each search area compared with the distribution expected by chance. The first positions, where most genes are found, are significant.

The E-Q method is among the most recent and efficient methods to compare phenotypes between different species (29,30). To evaluate its accuracy in Manteia, we ran a simulation on all OMIM diseases for which an HPO description was available and for which a gene was already suspected or known to lead to the disease (∼3100 OMIM gene–disease associations). About 28% of cases could not be analyzed mainly owing to a lack of phenotype annotation for the corresponding mouse genes. For the 2241 remaining gene–disease associations, Figure 6a shows that 387 genes are accurately found within the first 50 candidates when only 16 are expected by chance. Even though this method can give excellent results, this simulation shows that for many cases, the clinical features alone are not enough to predict accurately the right candidate genes. The algorithm cannot distinguish the right candidate among genes responsible for similar phenotypic features in the genome.

To overcome this problem, Manteia allows the investigators to narrow down the number of candidates by using this automatic approach along with their own evidences. For example, one can constrain the search to a chromosomal location given by a CGH array or a linkage analysis using a combination of the ‘chromosome location’ and ‘OMIM’ tools. For example, most DiGeorge syndrome cases result from a microdeletion on chromosome 22q11.2. To prioritize the candidate genes potentially responsible for the disease, one can enter these coordinates in the system, obtain the mouse orthologs using ‘Refine’ and then go to the DiGeorge page in the OMIM module to start the automatic search. *TBX1*, which has been proven to be associated to the disease (31), ranks first in the list (see Supplementary Table S3). Interestingly, Manteia ranks *CRKL* in second position. This gene plays a major role in the formation of the structures affected by the syndrome (32). Note that without using the chromosome localization data, *TBX1* ranks 17th among the candidates.

We further tested the benefits of using mapping information in the previous analysis by running simulations over different intervals around the disease gene (the size of the search area depends on the precision with which the disease locus has been mapped). Once again, all known disease genes from OMIM are searched in Manteia using the corresponding clinical description of the patients among the candidate genes contained in these chromosomal intervals. The position in which the known gene is found among the candidates measures the precision of the method. The results are then compared with the number of known genes expected by chance for each ranking position when picked randomly in the same areas of the genome (See ‘Materials and Methods’ section). Using the positional information, 94, 86, 57 and 46% of known genes rank within the first 10 positions in the prediction list when regions of 5, 10, 50 and 100 Mb are analyzed, respectively (Figure 6b). These results are highly significant (Figure 6c–f) and are considerably better than the E-Q method used alone (Figure 6b). The unique possibility offered by Manteia to combine automatic and manual search has clear value for human genetics where in many cases, the disease locus has not been identified but has been mapped to a specific linkage interval.

## DISCUSSION

Manteia is an original online resource that provides a cohort of data from different species with a set of powerful tools within a structured environment to unleash the potential of combinatorial large-scale analyses of biological and clinical data. The system is extremely versatile and can be used for many types of analyses, including data retrieval, gene or probe set annotation, information content analysis, candidate gene prediction and prioritization. The user interface has been designed to make all analyses easy and fast, and an online help is provided to familiarize the novice with the system. The system architecture is modular and will allow the addition of new animal models, new data sets and new tools to extend even more the scope of its applications.

This tool should prove particularly helpful for investigators interested in finding biologically significant correlations in large lists of genes and proteins generated by modern high-throughput approaches. Thanks to its ability to combine genomic, phenotype and medical data, Manteia is also particularly well adapted to help geneticists in investigating the genetic origin of human diseases, as shown with our simulations. Phenotype data are clearly the most valuable source of information for this kind of predictions. The relatively restricted amount of phenotype data currently available represents the most important limitation to this approach. However, the continual efforts made by the International Mouse Phenotyping Consortium aiming at describing the null mutations of every single gene from the mouse genome should help resolve this problem over time. Manteia predictions are computed live, which has several advantages. It allows the users to add their own information to the queries and it eases the maintenance of the system. Hence, new information can be exploited immediately in Manteia by updating its knowledge base. In this regard, we have designed an automated annotation pipeline to keep Manteia up-to-date. In this way, the information content of the system will continuously increase in size and quality to enhance the sensitivity of predictions made by the system.

## SUPPLEMENTARY DATA

Supplementary Data are available at NAR Online.

## FUNDING

Stowers institute for medical research; Howard Hughes medical institute (HHMI); ‘Institut national de la santé et de la recherche médicale’ (INSERM); European research council (ERC) [249931]; Cotrel foundation. Funding for open access charge: ERC.

*Conflict of interest statement*. None declared.
